# Mechanisms of Action of Ozone Therapy in Emerging Viral Diseases: Immunomodulatory Effects and Therapeutic Advantages With Reference to SARS-CoV-2

**DOI:** 10.3389/fmicb.2022.871645

**Published:** 2022-04-21

**Authors:** Alessandra Cenci, Iole Macchia, Valentina La Sorsa, Clemente Sbarigia, Valentina Di Donna, Donatella Pietraforte

**Affiliations:** ^1^Core Facilities, Italian National Institute of Health, Rome, Italy; ^2^Department of Oncology and Molecular Medicine, Italian National Institute of Health, Rome, Italy; ^3^Research Coordination and Support Service, Italian National Institute of Health, Rome, Italy; ^4^APS S.p.a., Rome, Italy; ^5^Rehabilitation Unit, IRCCS INRCA, Fermo, Italy

**Keywords:** ozone therapy, immune response, SARS-CoV-2, COVID-19, microbiome, antioxidant, cytokine storm, anti-inflammatory

## Abstract

Medical oxygen-ozone (O_2_-O_3_) is a successful therapeutic approach accounting on the assessed beneficial action of ozone in the range 30–45 μg/ml (expanded range 10–80 μg/ml according to different protocols), as in this dosage range ozone is able to trigger a cellular hormetic response *via* the modulating activity of reactive oxygen species (ROS), as signaling molecules. The ozone-dependent ROS-mediated fatty acid oxidation leads to the formation of lipid ozonization products (LOPs), which act as signal transducers by triggering ROS signaling and therefore mitohormetic processes. These processes ultimately activate survival mechanisms at a cellular level, such as the Nrf2/Keap1/ARE system activation, the AMPK/FOXO/mTOR/Sir1 pathway and the Nrf2/NF-kB cross talk. Furthermore, indirectly, *via* these pathways, LOPs trigger the HIF-1α pathway, the HO-1 signaling and the NO/iNOS biochemical machinery. Ozone-driven shift of cytokine activation pathways, from pro-inflammatory to anti-inflammatory immediately afterwards, also exert direct immunoregulatory effects on regulatory T lymphocytes as well as on the intestinal microbiota, which in turn can affect immune response thus influencing the progression of the disease. In this review, we will describe the biological and biochemical mechanisms of action of ozone therapy with the aim of evaluating both positive and critical aspects of ozone use as a therapeutic adjuvant in the light of emerging viral infections, such as SARS-CoV-2 and microbiome-associated disorders related to SARS-CoV-2.

## Introduction

Ozone (O_3_), a triatomic molecule consisting of three oxygen atoms, is commonly known as an O_2_-derived reactive gas generated in the different layers of atmosphere, mainly during storms through the action of electrical discharges and the ultraviolet light. However, while stratospheric ozone forms naturally and it helps to shield us from ultraviolet radiation in sunlight, the increasing concentration of this gas in the troposphere is determined by nitrogen oxides and volatile organic compound pollution.

The medical therapeutic potential of O_3_ (hereafter referred as O_2_-O_3_ therapy, where O_2_ must be used as the O_3_ generator) has been proposed since 1896, when Nikola Tesla built the first O_3_ generator device used as disinfectant as well as for medical therapy (Mandhare et al., 2012). Due to its versatility, this technique had been successfully used during the First World War to treat infections caused by several etiological agents, thanks to the ability of ozone to inactivate bacteria, some types of viruses, fungi, yeast, and protozoa (Khadre et al., 2001).

Medical ozone used in the O_2_-O_3_ therapy has nothing to be shared with the activity of ozone against microbes, as in the first case ozone exerts its action indirectly *via* the hormetic-mediated activity of molecules such as lipid ozonization products (LOPs), whereas in the second case ozone can directly kill viruses and bacteria, acting as a chemical toxicant in indoor and environmental spaces. In this respect, it is noticeable to mention some evidence regarding ozone as an anti-microbial agent against bacteria, both *in vitro* on Gram-positive and Gram-negative bacteria (Giuliani et al., 2018) and *in vivo* (Franzini et al., 2016), and against coronavirus-2 (SARS-CoV-2; Albert et al., 2021; Criscuolo et al., 2021; Percivalle et al., 2021). This property should enable ozone to be considered as a new therapeutic tool to be used in the fight against antibiotic-resistance. The mechanism of killing bacteria by ozone is complex because ozone attacks numerous cellular constituents until bacteria loose its shape (Giuliani et al., 2018). In 1980, O_2_-O_3_ therapy was proved to have multiple effects, such as the ability to induce anti-inflammatory and hemodynamic responses through the use of autohemotransfusion (AHT) with an O_2_-O_3_ mixture by German physicians (Stoker, 1902). During AHT, or Large Auto Emo Infusion with Oxygen-Ozone (GAEI), blood is removed and then reinjected intravenously into the patient, after having been ozonated (Bocci et al., 2001; Sagai and Bocci, 2011). Ozone therapy can also be performed by other techniques such as rectal insufflations or, in laboratory animals, also by peritoneal injection (Chirumbolo et al., 2021c). To avoid possible dangerous side effects, the protocols in medical ozone must be standardized. The Italian Society of Oxygen Ozone Therapy (SIOOT) assayed, optimized and improved such protocols, and approved the only standardized protocol for the use of ozone as a medical treatment (Chirumbolo et al., 2021a,b; Varesi et al., 2021). The O_2_-O_3_ therapy is nowadays a non-invasive medical practice free of side effects, applied in the treatment of several diseases, such as cardiovascular, neurodegenerative, orthopedic, dermatological, gastrointestinal, and genitourinary pathologies (Sagai and Bocci, 2011; Smith et al., 2017; Braidy et al., 2018; Scassellati et al., 2020). It is now well established that the unexpected beneficial effects of O_3_ in biological tissues are related to its ability to stimulate the intracellular signaling and metabolism, to boost the tissue antioxidant and anti-inflammatory activities and to potentiate the immunological response. The administration protocol, as well as the treatment concentration of O_3_, seems to be crucial for its therapeutic response, with low doses favoring a mild tissue oxidative stress in mitochondria (mitohormesis), leading to the activation of antioxidant adaptive and survival responses, whereas high doses favoring the occurrence of known damaging effects (Bocci et al., 2011; Chirumbolo et al., 2021b).

Ozone generators produce ozone through a high-voltage gradient (5–13 mV), starting from pure oxygen up to obtain a gaseous mixture containing a minimum of 95% oxygen and up to 5% ozone. As an example, the 50 μg/ml treatment used for medical purposes is composed of 97.5% oxygen and 2.5% ozone. Usually, for clinical purposes, ozone concentrations range is 30–45 μg/ml. In most cases, an ozone dose lower than 10 μg/ml of gas per ml of blood is biologically ineffective because ozone is totally neutralized by plasma antioxidants, whereas doses higher than the therapeutic threshold can become toxic (Bocci, 2006a). In the exposition of blood to O_2_-O_3_, the extra oxygen is to convey and chemically stabilize O_3_, having a negligible therapeutic value, because it rapidly equilibrates within the blood vessels in both the extracellular and intraerythrocytic water before becoming bound to hemoglobin, which became fully oxygenated (Bocci et al., 2009).

An important application of O_2_-O_3_ therapy concerns viral diseases. Through their interaction with viral capsid, the O_3_-derived LOPs have been reported to induce the peroxidation of the glyco-proteins and lipids, leading to a weakness of lipids coated virus and to a drastic reduction of the viral load (Murray et al., 2008; Ataei-Pirkooh et al., 2021). However, as reported below, the major mechanism of ozone pharmacological antiviral activity is linked to the ability of ROS and LOPs to induce, at low doses, a mild intracellular and mitochondrial oxidative stress-mediated signaling able to trigger an antioxidant, anti-inflammatory, anti-thrombogenic response (Chirumbolo et al., 2021c). These processes, called hormesis and mitohormesis, respectively, hesitate in the reduction of organ damage in ischemia/reperfusion injury models, the endothelial damage, damping the thrombotic mechanisms and favoring the cardiovascular protection (Chirumbolo et al., 2021c). It is clear that the O_2_-O_3_ therapy appears therefore to be a powerful tool also against the severe acute respiratory syndrome coronavirus 2 (SARS-CoV-2), the coated pathogenic virus responsible for the recent widespread COVID-19 pandemic (Martínez-Sánchez et al., 2020; Chirumbolo et al., 2021c). The SARS-CoV-2 infection boosts the innate and adaptive immunity, inducing a strong inflammatory status, oxidative stress and tissue damage and triggers a cardiovascular dysregulation characterized by intravascular coagulation and thrombosis (Amor et al., 2020).

In this review we will describe the principal ozone pathways as well as anti-inflammatory and immunomodulatory pathways both having cytoprotective effects. In particular the anti-inflammatory effects due to ozone are mediated by inhibition of B4 leukotrienes and NF-kB expression consequent to NRF2 activation and NLRP3 inhibition. Activation of NRF2 has an important effect on NF-kB inhibition and consequently on inflammatory cytokines expression linked to NF-kB activity. It also affects NLRP3 inhibition by hindering the formation of the inflammasome. Of note is the protective role of ozone in cardiomyocytes, since it has been described a possible pathophysiological role for SARS-CoV-2 in related myocarditis (Siripanthong et al., 2020). Simonetti et al. (2019) describes for the first time a protective role of ozonotherapy in cardiomyocytes and fibroblasts treated *in vitro* with doxorubicin, a chemotherapeutic drug used to treat cancer. The increase in IL-1 related to hs-CRP (High Sensitivity C-reactive protein), a protein linked to a cardiovascular risk factor, together with the increase in free radicals is among the trigger points of toxicity induced by doxorubicin, in addition to the alteration of intracellular calcium homeostasis. In this work in which human fetal cardiomyocytes and human fibroblasts were treated with doxorubicin alone or in combination with increasing ozone concentrations (from 10 to 50 μg/ml), ozone treatment significantly reduced the expression of inflammatory cytokines and inflammatory mediators in both cell types. Another cytoprotective effect was due to NRF2 activation observed by using an ozone concentration of 30 μg/ml up to a plateau phase at higher concentrations.

Of particular interest is the ability of lipid-derived O_3_-dependent oxidant species, such as oxysterol, to inhibit SARS-CoV-2 infection (Zu et al., 2020).

In this review, we will summarize the biochemical and biological mechanisms at the basis of the O_2_-O_3_ therapy and explain how ozone and its derivatives boost the antioxidant and immune-stimulating processes thus giving a glimpse of new perspectives of this therapy in emerging human viral and microbial diseases, including COVID-19 and microbioma-associated disorders (Hernández et al., 2020; Rowen and Robins, 2020).

## A Mechanistic Insight

**Figure 1:**
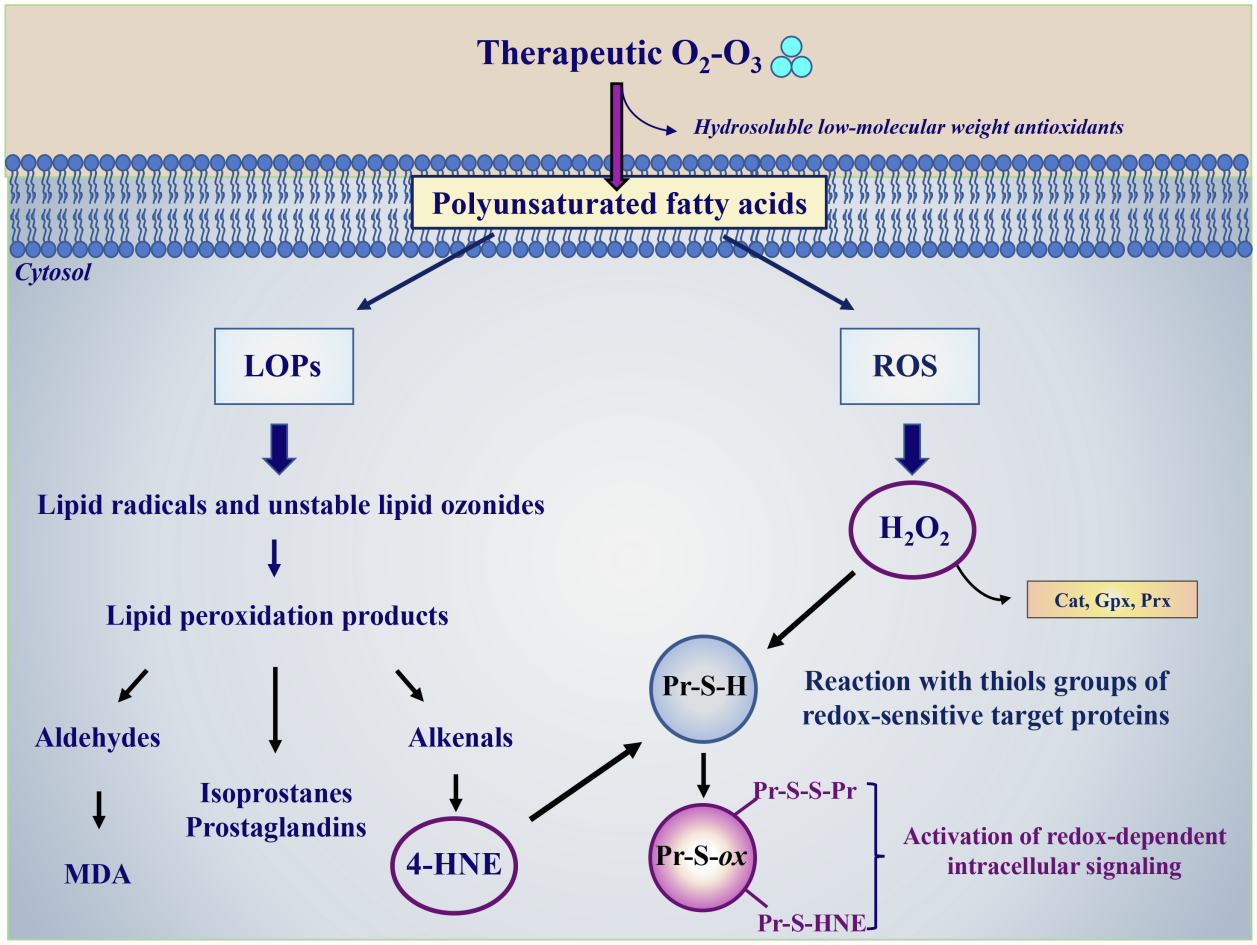
Ozone therapy as a modulator of the redox intracellular signaling and tissue antioxidant capacity. O_3_ dissolves very rapidly in biological fluids where it can react with hydrosoluble low molecular weight antioxidants (ascorbic acid, uric acid, and glutathione) or with biological membranes. In its reaction with polyunsaturated fatty acids, O_3_ gives rise to multifaceted reactions generating lipid ozonization products (LOPs) and reactive oxygen species (ROS), with H_2_O_2_ being the most abundant species. LOPs include lipid radicals, unstable lipid ozonides and lipid peroxidation products, such as aldehyde among which malondialdehyde (MDA), isoprostanes, prostaglandins and alkenals, with 4-hydroxynonenal (4-HNE) having a key role in the biological activity of O_3_. The overloading of intracellular H_2_O_2_ amounts is avoided by the detoxifying activity of specific antioxidant enzymes, such as catalase (Cat), glutathione peroxidase (Gpx), and peroxiredoxin (Prx). 4-HNE and H_2_O_2_ represent the most relevant secondary messengers mediating the ozone therapy beneficial effects. 4-HNE and H_2_O_2_ oxidize critical thiols groups of redox-sensitive target proteins (Pr-S-H) inducing their oxidation (Pr-S-*ox*) with the formation of reversible disulfides bridges with other thiol groups (Pr-S-S-Pr) or covalent irreversible adducts (S-HNE), respectively. These thiol modifications alter the targets’ structure and activity and the redox-regulated processes linked to the involved modified enzymes, activating the intracellular signaling and triggering the expression of cytoprotective and survival genes aimed at enhancing the cellular protection against oxidative stress.

O_3_ possesses highly solubility in the presence of H_2_O (10-fold more soluble than O_2_) allowing this gas to dissolve very rapidly in biological fluids (Battino et al., 1983). This property of O_3_ limits its wide diffusion in biological tissues and allows the molecule, or its major oxidative byproduct hydroxyl radical (^•^OH), to react immediately with hydrosoluble antioxidants (ascorbic acid, uric acid, glutathione) and lipids (Pryor et al., 1985; Bocci et al., 2011; Figure 1). The hydrosoluble antioxidants detoxify part of the O_3_ through a sacrificial protection mechanism that leads to production of few bioactive products (Pryor et al., 1995). The reaction of O_3_ or its derived reactive species with lipids is of particular importance in those tissues where the concentration of these macromolecules is appreciable, such as in alveolar and small airway lining fluid in which they represent about 90% of tissue components (Pryor et al., 1985) or in blood (Ueno et al., 1998; Bocci, 2011). Among lipids, polyunsaturated fatty acids (PUFA) represent the main O_3_ target generating specific LOPs and H_2_O_2_ (Figure 1). In the absence of H_2_O, O_3_ also reacts with PUFA producing Criegee ozonide, but this reaction is not favored in biological tissues where H_2_O is highly abundant. LOPs have a long-life span, before they reach the vascular wall and interact with tissues where they trigger late effects. On the contrary, H_2_O_2_ has a shorter life-span and it is responsible for the first biological effects on blood cells (erythrocytes, leukocytes, and platelets) where it modulates the activity of sensible redox-related targets by modulating the intracellular signaling (Figure 1).

It is fundamental to underline again that the beneficial therapeutic effects of O_2_-O_3_ therapy are based on the ozone ability to act through a hormetic dose–response mechanism, being able to promote the generation of biochemical mediators capable of inducing beneficial mild oxidative stress effects (Bocci et al., 2011; Galiè et al., 2019). The beneficial mild oxidative stress, induced when administrated in the threshold of 30–45 μg/ml, the therapeutic O_2_-O_3_ window (Travagli et al., 2007), is elicited by the production of controlled low doses of ROS mainly acting as signaling molecules and boosting the formation of LOPs. This behavior makes medical ozone similar to other gases of physiological importance, such as NO, CO, and H_2_S (Kolluru et al., 2017). These molecules are known for their ability to act as signaling and antioxidant molecules of potentially therapeutic application, although, contrary to medical ozone, they could become pro-oxidants and promoters of redox status alteration and intracellular oxidative damage when high concentrations are achieved in the tissues. In response to the ozone-induced mild oxidative stress, mitochondria membrane potential undergoes modification favoring the generation and release of low levels of ROS, a process linking mitochondria to the nucleus known as mitochondrial hormesis or mitohormesis (Ristow, 2014; Bárcena et al., 2018). As a retrograde signaling molecules, mitochondrial-released ROS induce the activation of intracellular redox-related signaling pathways, such as those involving NRF2/Keap1, forkhead box O proteins (FoxOs), heat shock factors, or antioxidant enzymes such as peroxiredoxin 2, promoting an adaptive response aimed at protecting the cells from further damage (Ristow, 2014; Bárcena et al., 2018). The adaptive responses, which are the fundamental basis of defense strategies, include the activation of mechanisms leading to increase the expression of (i) antioxidant and phase I/II detoxifying enzymes, (ii) macromolecules improving mitochondrial homeostasis, such as mitochondrial unfolded protein response which repairs or clears misfolded proteins, (iii) chaperons HSPs, involved in protein homeostasis and powerful stimulators of innate immune response, (iv) the uncoupling protein 1, (v) the unique mitochondrial membranous protein devoted to adaptive thermogenesis, and (iv) to modify the intracellular metabolism due to the production of mitochondrial metabolites, such as ATP, NAD^+^, calcium, acetyl-CoA, leading to an improve in health and viability within a cell, tissue, or organism (Ristow, 2014; Bárcena et al., 2018).

The next two paragraphs will analyze in depth the mediators involved in O_3_ beneficial effects.

## O_3_-Derived H_2_O_2_ and Cellular Redox Signaling

The main ROS produced by O_3_ during the reaction with targets is the non-radical species H_2_O_2_ (Figure 1). In general, in biological systems the H_2_O_2_ transport inside cells is mainly mediated by diffusion, by changes in membrane lipid composition or by aquaporin proteins channels AQP3 and AQP8 (Bienert et al., 2006). H_2_O_2_ acts through a hormetic dose–response-mediated mechanism. At low concentrations (about 0.5–0.7 μM), H_2_O_2_ is sensed and used as a redox signaling molecule able to drive different biological processes such as cell growth, migration, differentiation, apoptosis as well as the expression of antioxidant enzymes (Stone and Yang, 2006). The mechanism behind H_2_O_2_-mediated physiological redox signaling regulation implies its interaction with key signaling proteins and involves the reversible oxidation of cysteine residues present in catalytic enzymes or regulatory sites, as well as in receptors, channels and transporters, transcription factors, kinases, and phosphatases (Stone and Yang, 2006; Janssen-Heininger et al., 2008). These effects, detectable at low peroxide concentration, are at the basis of the beneficial effects of O_2_-O_3_ therapy. The toxic effects of H_2_O_2_ occur when its concentration increases. Indeed, H_2_O_2_ may: (i) irreversibly inhibit the activity of proteins through the over-oxidization of the critical cysteine residues leading to the dysregulation of the intracellular redox signaling and (ii) react with metal-centers present in peroxidases and hemoproteins, favoring the Fenton reaction-driven formation of strong reactive oxidizing species, such as ^•^OH, and metal-centered ferryl species. These strong oxidants are able to oxidize target molecules, allowing further protein dysfunction and alteration in cell signaling.

The effects of H_2_O_2_ on cell signaling lead to the activation of the inflammasome and the redox-sensitive transcription factors like the inducible hypoxia factor type 1 alpha (HIF-1α) and the nuclear factor kβ (NF-kβ), with the production of pro-inflammatory cytokines IL-1β, IL-6, and TNF-α (Mittal et al., 2014). Once in the cytoplasm, H_2_O_2_ activates indeed the IKappaBeta tyrosine kinase (IKK) which phosphorylates the IkB fraction of NF-kβ complex, allowing the heterodimer p50-p65 to move to the nucleus. Here, the heterodimer p50-p65 regulates gene expression of different types of proteins such as the isoform 2 of the pro-inflammatory enzyme cycloxigenase (COX), the nuclear factors of activated T cells (NFAT) and the activator protein 1 (AP-1) heterodimer (Fos-Jun; Chen et al., 1998). These proteins give rise to the activation of different pathways, such as the activation of GPC6 expression and WNT5A signaling pathway and the production of the pro-inflammatory cytokines IL-2, IL-6, IL-8, TNF-α and IFN-γ leading to an inflammatory milieu that will recruit neutrophils, lymphocytes and macrophages (Sen and Baltimore, 1986; Li and Verma, 2002). In addition, H_2_O_2_ activates the node-like receptor protein (NLRP3) that belongs to a protein complex called inflammasome (Surabhi et al., 2021). It is important to remind that the ozone concentrations suitable to induce the above-mentioned H_2_O_2_ damaging effects are never achieved in O_2_-O_3_ therapy, being damaging to functional and crucial proteins for the living host. Relatively high H_2_O_2_ concentration can be locally achieved under inflammatory conditions characterizing a pathological status or refers to the direct activity of ozone used to neutralize bacteria and viruses outside the organisms and *in vitro* models.

Under inflammatory conditions, a small quantity of O_3_, H_2_O_2_, and dihydrogen trioxide was assumed to be produced during infections through the ^1^O_2_-driven water oxidation catalyzed by antibodies bound to neutrophils (Wentworth et al., 2002; Babior et al., 2003). Neutrophils, indeed, have been reported to bind antibodies, and once activated by an exogenous stimulus, to produce a species with the chemical signature of O_3_ through the ^1^O_2_-mediated oxidation of water. However, the occurrence of this process, proposed as a neutrophil activation mechanism involved in pathogens killing during inflammation, remains highly controversial. Taking into account the low H_2_O_2_ levels produced in this process, the high reactivity of the produced species and, more importantly, their fast reaction with the low molecular weight antioxidants preventing reactions with other biological targets, the antibody-mediated production of H_2_O_2_, O_3_ and dihydrogen trioxide hardly occur *in vivo* (Pryor et al., 2006).

## O_3_-Derived LOPs Biological Activity

**Figure 2:**
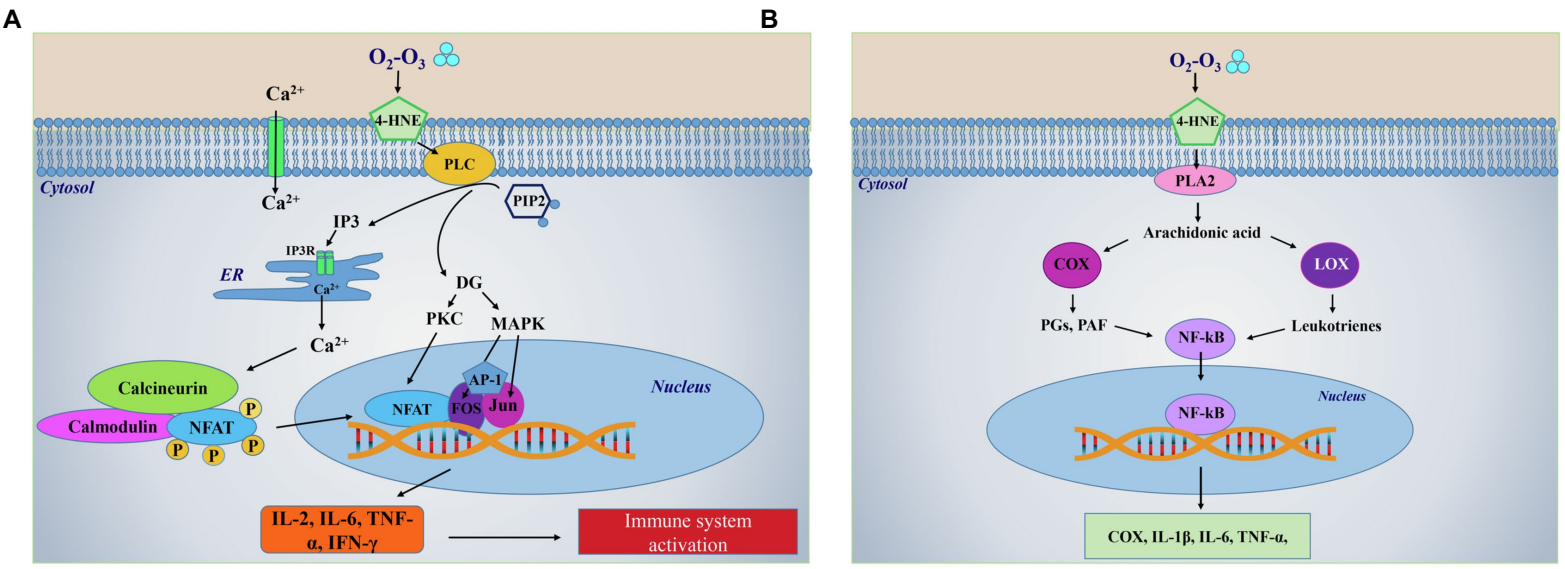
LOPs-dependent activation of lipids-related intracellular pathways. **(A)** Ozone is highly unstable and reacts in milliseconds with biological fluids by oxidizing lipids producing lipid ozonization products LOPs mechanism of LOPS-mediated immune system activation through the activation of phospholipase-linked pathway. LOPs, in particular 4-HNE, transduce the cell signal by activating phospholipase C (PLC) which hydrolyzes a membrane lipid phosphatidylinositol l-4,5 bisphosphate (PIP2) to produce second messengers, i.e., inositol triphosphate (IP3) and diacylglycerol (DG). IP3 binds to the inositol triphosphate receptor (IP3R) on the endoplasmic reticulum (ER) membrane, resulting in a release of Ca^2+^ from the ER to the cytosol. Increased levels of Ca^2+^ in the cytosol activate calcineurin, a Ca^+2^/calmodulin-dependent phosphatase which acts dephosphorylating the nuclear factors of activated T cells (NFAT) and transports them to the nucleus. Here, NFAT then induces transcription of cytokines, such as IL-2, IL-6, TNF-α, and IFN-γ triggering the first inflammatory response that precedes immunoactivation. In parallel, hydrolysis of PIP2 by PLC results in production of DG, which activates a protein kinase C (PKC) and the mitogen-activated protein kinases (MAPK). The activation of these kinases leads to synthesis and activation of Fos and Jun, the components of the heterodimeric transcription factor activator protein 1 (AP-1). Then, AP-1 complex binds cooperatively with NFAT to composite NFAT:AP-1 sites in the regulatory regions to activate transcription of NFAT target genes, which carry to the synthesis of pro-inflammatory cytokines. **(B)** Mechanism of LOPS-mediated synthesis of pro-inflammatory mediators through the activation of phospholipase A2-linked pathway. 4-HNE can induce the synthesis of arachidonic acid through the activation of phospholipase A2 (PLA2). Arachidonic acid can be converted in prostaglandins (PGs) and platelet activating factor (PAF) *via* the activation of cyclooxygenase (COX), or in leukotrienes *via* the activation of lipoxygenase (LOX). These compounds induce the transcription of the nuclear factor kappa-light-chain-enhancer of activated B cells (NF-kB), whose translocation into the nucleus, allow the further synthesis of the pro-inflammatory COX or cytokines (IL-1β, IL-6, and TNF-α). Part label **(A)** is modified from https://www.tebu-bio.com/blog/focus-on-the-calcineurin-nfat-pathway/.

LOPs are generated through multiple ruptures of PUFA subsequent to O_3_-mediated oxidative reactions (Pryor et al., 1995). These short-chain lipoperoxides loose in part the hydrophobic characteristic of lipids and become water-soluble, a property that allows them to be transported into the plasma. LOPs are considered the species most likely acting as signal transduction compounds and include lipid-centered radicals, hydroperoxides, isoprostanes, ozonides, aldehydes and alkenals (Figure 1). Among alkenals, 4-Hydroxynonenal (4-HNE) or 4-hydroxy-2-hexenal, depending on whether they derive from Ω 6 or Ω 3 fatty acids, behave as second messengers inside cells, since ozone is unable to cross the cell membrane. 4-HNE is the most abundant product generated in the reaction and it is the bioactive lipid peroxidation product able to react with all principal biological molecules such as proteins, lipids, nucleic acids (Schaur et al., 2015). With regard to the activation of the redox-dependent intracellular signaling, the 4-HNE mechanism of action is similar to that shown for H_2_O_2_, i.e., it is based on its binding to thiols groups of redox-sensitive target proteins (Schaur et al., 2015; Figure 1). The formation of these complexes causes the target proteins to be activated and to translocate from cytoplasm to the nucleus where they function as transcription factors. Among these, Nrf2, NF-Kβ and inducible HIF-1α are the most studied (Larini and Bocci, 2005; Schaur et al., 2015). Cholesterol may represent another important target of O_3_ generating bioproducts known as oxysterols, which are able to induce cell activation and cytokine release, functioning as immuno-modulators (Choi and Finlay, 2020). LOPS can act locally or systemically in all cells of the organism including both epithelial and endothelial cells as well as in macrophages and other immune cells. LOPs are known to activate specific lipases, such as phospholipase C (PLC), and phospholipase A2 (PLA2; Figure 2). In particular, activated PLC hydrolyzes the membrane lipid phosphatidylinositol-4,5 bisphosphate (PIP2), to produce second messengers, i.e., inositol triphosphate (IP3) and diacylglycerol (DG). IP3 binds to the inositol triphosphate receptor (IP3R) on the endoplasmic reticulum (ER) membrane, resulting in a release of Ca^2+^ from the endoplasmic reticulum to the cytosol (Figure 2A). Increased levels of Ca^2+^ in the cytosol activate calcineurin (CN), a Ca^+2^/calmodulin-dependent phosphatase, which acts dephosphorylating NFAT and transports them to the nucleus. NFAT then induces transcription of cytokines such as IL-2, TNF-α, IL-6 and IFN-γ and other immune responses (Wang et al., 2007). In addition, arachidonic acid (AA), produced by the LOPs-mediated activation of PLA2 can be converted in prostaglandins (PGs) and platelet activating factor (PAF) *via* COX and lipoxygenase (LOX) leading to the production of the pro-inflammatory cytokines TNF-α and IL-6 *via* the transcription factor NF-Kβ (Braidy et al., 2018; Figure 2B). In the case of medical O_2_-O_3_ therapy, the hormetic behavior of LOPs at low concentration favors the reduction of inflammation and the promotion of cell survival. Through the inhibition of pathway linked to p38MAPK and ERK1/ERK2 signaling, 4-HNE can down-regulate the synthesis of pro-inflammatory cytokines (TNF-α, IL-1β) from LPS-stimulated monocytes (Marantos et al., 2008). In addition, 4-HNE has been reported to stimulate the synthesis of anti-inflammatory (γ-glutamyl transferase, γ-glutamyl transpeptidase, HSP-70, HO-1), and antioxidant compounds (SOD, GPx, CAT and glucose-6-phosphate dehydrogenase; Clavo et al., 2018). Moreover, the LOPs cyclopentenone isoprostanes behave as negative feedback regulators of the inflammatory response in LPS-activated macrophages by inhibiting the degradation of IkBα and subsequent nuclear translocation and transcriptional activity of NF-kB, as well as the expression of inducible NOS and COX-1 inhibiting the pro-inflammatory status (Musiek et al., 2005). Furthermore, the reaction of LOPs with Keap1 thiol groups reduces the proteasome-mediated degradation of Nrf2 favoring its activation, and consequently inducing the activation of survival genes through the inhibition of the apoptotic pathways mediated by both caspase 8-linked signaling and mitochondria (Groeger and Freeman, 2010). The LOPs-mediated activation of NRf2, through the Keap/ARE axes, triggers the synthesis of HO-1 and contributes to inhibit platelets-dependent thrombosis (Lindenblatt et al., 2004; Peng et al., 2004).

As antiviral agents, LOPs can further boost the release of heat shock proteins (HSP), such as HSP60, HSP70 and HSP90, since these proteins are powerful stimulators of the innate immune response. In particular these proteins are able to induce cytokines release from the monocyte–macrophage system and the activation of the antigen-presenting cells (Bocci, 2011; Sagai and Bocci, 2011).

## Antioxidant Effect of O_3_

The first O_3_ antioxidant effects were explored by the studies of Bocci’s group showing that, by using doses below the threshold of the O_2_-O_3_ therapeutic window, i.e., below the concentration of 80 μg/ml, whole blood was protected from hemolysis and its reactivity was controlled for about 20%–40% by the antioxidant system of the plasma, avoiding the toxic effects observed at higher concentrations, such as those detected in erythrocytes (lipid peroxidation, methemoglobin levels, hemolysis; Travagli et al., 2007; Bocci et al., 2009).

**Figure 3:**
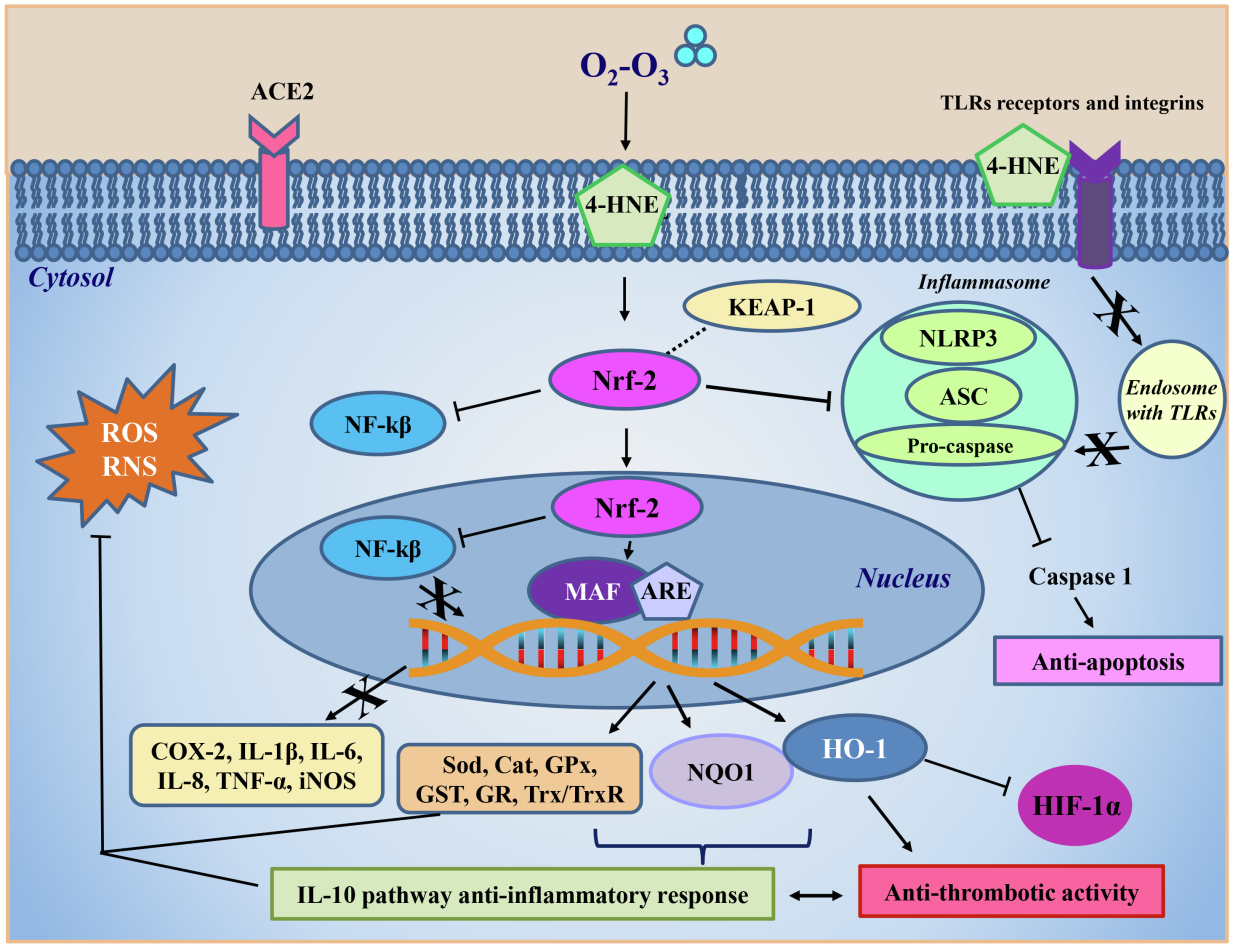
Ozone antiviral intracellular mechanism. The 4-HNE formed following ozone (O_2_-O_3_) treatment interacts in the cytoplasm directly with the nuclear factor erythroid 2-related factor 2 (Nrf-2), which freed from inhibition with the Kelch-like ECH-associated protein 1 (Keap-1), inhibits the activity of the nuclear factor kappa-light-chain-enhancer of activated B cells (NF-kβ) also after its translocation into the nucleus. Here, Nrf-2 acts as a second messenger binding to the musculoaponeurotic fibrosarcoma transcription factor (MAF) and to the antioxidant responsive elements (ARE) regions. This process allows transcription of antioxidant enzymes such as superoxide dismutase (Sod), catalase (Cat), glutathione peroxidase (Gpx), glutathione-S-transferase (GST), glutathione reductase (GR), and the system thioredoxin/thioredoxin reductase (Trx/TrxR), NADPH quinone oxidoreductase 1 (NQO1), and hemeoxygenase (HO-1), all aimed at counteracting reactive oxygen (ROS) and nitrogen (RNS) species overproduction and restoring the physiological redox balance. The formation of the complex Nrf2/MAF/ARE also results in the inhibition of the transcription of pro-oxidant enzymes, such as cycloxygenase (COX) and the inducible isoform of the nitric oxide synthase (iNOS), or pro-inflammatory cytokines, such as IL-1β, IL-6, IL-8, and TNF-α. NQO1 is a detoxificant enzyme that protects cells from oxidative stress by lowering reactive oxidizing species, protecting DNA, proteins and lipids from hyperoxia damage. HO-1 synthesis is boosted by the 4-HNE-mediated increase of Nrf2 levels. HO-1 lowers oxidative stress by inhibiting the high level of expression of HIF-1α, a molecular O_2_ sensor which is activated during inflammation, and protecting epithelium damage from apoptosis and thrombosis by downregulating platelet activation. Through the activation of Nrf2, 4-HNE inhibits the activity of the inflammasome, a pro-inflammatory complex formed by the nucleotide-binding domain leucine-rich repeat and pyrin domain containing receptor 3 (NLRP3), the adaptor apoptosis-associated speck-like protein (ASC) and the caspase 1 effector protein. The inflammasome inhibition blocks the activation of caspase 1 and inflammatory interleukins, favoring in turn the anti-inflammatory cytokines production and consequently the downregulation of the apoptotic pathways. The IL-10 pathway anti-inflammatory response, stimulated by the ozone antioxidant reaction, contributes to the decrease of the thrombotic activity and prevents vascular injury.

A central O_3_-mediated antioxidant role was played by the up-regulation of Nrf2, widely known as a key regulator of several cytoprotective responses, including the expression of fundamental ROS-detoxifying enzymatic systems (Figure 3). Under normal conditions, Nrf2 is expressed at low levels, and it is found in the cytoplasm linked to its specific Keap1 inhibitor that promotes its degradation through the ubiquitin pathway (Itoh et al., 1999; Bocci et al., 2009). O_3_ has been reported to activate Nrf2 in a dose-dependent manner (Galiè et al., 2019) and, although this mechanism still needs to be clarified, the O_3_-derived alkenals have been shown to dissociate the Nrf2-Keap1-BCR complex by activating Nrf2 (Figure 3; Janssen-Heininger et al., 2008; Li et al., 2008). Keap1 has two -SH groups which undergo LOPs-mediated oxidation changing protein conformation by releasing Nrf2. The protein thus accumulates in the nucleus, where it interacts with the MAF transcription factor forming a heterodimer that binds to the DNA regions containing the antioxidant response elements (ARE). This binding induces the transcriptional activation of more than 200 genes with antioxidant action such as superoxide dismutase (SOD), catalase (CAT), glutathione peroxidase (GPx), glutathione reductase (GR), glutathione-S-transferase (GST), NADPH quinone oxidoreductase 1 (NQO1), hemeoxygenase (HO-1), and the thioredoxin (Trx)/thioredoxin reductase (TrxR) system (Re et al., 2014; Galiè et al., 2019). The action of these antioxidant enzymes counteracts that of inflammation-linked pro-oxidant enzymes such as NADPH oxidase, NOS, xanthine oxidase, lipoxygenase, COX, and myeloperoxidase (MPO). These pro-oxidant enzymes are known to play a key role under inflammatory conditions characterized by the activation of vascular wall endothelial cells, vascular smooth muscle cells, fibroblasts and blood cells (including phagocytic cells, platelets and erythrocytes). These enzymes produce and release high levels of oxygen- and nitrogen-derived free radicals (O_2_^•^, ^•^OH, ^•^CO_3_, ^•^NO_2_) and non-radical reactive species (H_2_O_2_, ONOO^−^; Janssen-Heininger et al., 2008; Mittal et al., 2014). These species, which are produced by phagocytes during inflammation, are potentially able to induce irreversible cell damage through the posttranscriptional modification of intracellular signaling-linked proteins (Janssen-Heininger et al., 2008; Mittal et al., 2014). Hypochlorite (HClO) formed by MPO released by activated phagocytes can oxidize plasma lipoproteins and favor their binding to the specific receptors on platelet surface, then indirectly favor the proinflammatory and procoagulant platelet functions. The already mentioned ARE-controlled enzymes contribute to restore the intracellular redox homeostasis altered by the inflammation-derived oxidative stress by (i) scavenging the over-produced oxidants, (ii) restoring the levels of low molecular weight antioxidants (glutathione, biliverdin), (iii) maintaining the thiol groups in their reduced form suitable for cell signaling, and (iv) promoting the reduction of ROS-generating compounds, such as quinones. In particular, NQO1 is a detoxificant enzyme that protects cells from oxidative stress by lowering ROS, protecting DNA, proteins and lipids from hyperoxia-mediated damage (Burke et al., 2021).

Most of these antioxidant enzymes not only eliminate the free radicals produced by viral infection, but also contribute to decrease cytokine-mediated inflammation through the induction of leukotriene B4 reductase, a NAD(P)H-dependent oxidoreductase which inactivates leukotriene B4 involved in the inflammation and reduces oxidative stress counteracting the iron overload by increasing the ferritin levels (Dick et al., 2001). These processes could favor the mechanisms of protection from apoptosis that had been induced by oxidative stress. In particular, the increased levels of HO-1 induced by Nrf2 protect cells from apoptosis, defending cells against oxidative stress, because HO-1 inhibits thrombosis mechanism in downregulating platelet-dependent arterial thrombosis (Peng et al., 2004). Moreover, Nrf2 is also involved in the stimulation of DNA repair mechanisms, mediated by p21 (Villeneuve et al., 2009). Nrf2 in steady state is inactivated by Keap1 ligand or ubiquinated, whereas in the presence of p21, Nrf2 ubiquination is prevented. In this way, p21 is able to induce cell-cycle arrest between G1 and S phase, permitting DNA repair (Sun et al., 2020). At the same time the activation of Nrf2 pathway has a role in the repair and removal of damaged proteins through the direct recognition of SOS proteins (Li et al., 2008).

## Anti-inflammatory and Immunomodulating Effects of O_3_ in Cells and Tissues

In addition, Nrf2 can suppress NF-kβ activity also by the direct protein–protein interactions, during moderate oxidative stress (such as the one induced by O_3_; Wardyn et al., 2015; Kobayashi et al., 2016; Mohan and Gupta, 2018; Martínez-Sánchez et al., 2020).

Another immunomodulating key action of O_3_ consists in the inhibition of the NLRP3 (Wang et al., 2018; Figure 3). NLRP3 is an intracellular sensor belonging to the inflammasome complex, primarily expressed in the cytosol of innate immune and inflammatory cells, such as circulating monocytes, tissue macrophages, dendritic cells and neutrophils. NLRP3 is able to recognize various signals in response to microbial attack, such as molecules with conserved motifs that are associated with pathogen infection, the so-called pathogen-associated molecular patterns (PAMPs) inducing tissue damage, and danger-associated molecular patterns (DAMPs), activating and assembling the inflammasome. The inflammasome thus is a protein complex formed by NLP3, together with the adaptor apoptosis-associated speck-like protein (ASC protein also called PYCARD) and the caspase 1 effector protein (Swanson et al., 2019).

Caspase 1, once activated by ASC proteolytic cutting, give rise to a series of reactions resulting in the production of IL-1β and IL-18 that promote systemic inflammation. Recently it has been observed that the NLRP3 protein is expressed at high levels in progressive chronic kidney disease (CKD). Given that NLRP3 is essential for the inflammasome assembly it follows that it is an excellent target for CDK (Yu et al., 2017). In their papers, Yu et al. (2017) studied progressive CKD in an animal model, and observed that rats, treated with O_3_ therapy by rectal insufflation had a statistically significant reduction in the expression and production of the NLRP3 protein, ASC1 and caspase 1 compared to controls. Moreover, in O_3_-treated rats there was a decrease in the expression of IL-1β, TNFα, and IL-6 inflammatory cytokines as well as a glomerular improved morphology, thus demonstrating the anti-inflammatory action of O_3_. This paper may have important implications in SARS-CoV-2 infection since this virus activates the inflammasome complex by NLRP3 (Wang et al., 2018). In a study analyzing 124 patients with severe or moderate COVID-19 disease, it was shown that the magnitude of NLRP3 inflammasome activation in PBMCs *via* caspase 1 and IL-1 β production was related to the severity of clinical outcome (Rodrigues et al., 2021). The same mechanism is reported in a study on mice with ischemia–reperfusion (IRI) lesion, which is one of the main causes of pulmonary dysfunction during many pathological diseases including COVID-19, where O_3_ therapy gave similar results (Wang et al., 2018). In addition, Wang et al. (2018) showed that oxidative ozone treatment protects lungs from IRI by inhibiting the domain-like oligomerization of NLRP3, blocking the inflammasome complex, thus improving the antioxidant activity of Nrf2 and inhibiting apoptosis (Figure 3).

## Antiviral Action of O_3_

The antiviral action of O_3_ follows different pathways. As reported above, O_3_-derived intermediates are able to inactivate lipid-coated viruses through a peroxidation reaction, creating damages to both the lipid envelope and the protein shell (Murray et al., 2008; Ataei-Pirkooh et al., 2021). Vesiculoviruses belonging to the Rhabdoviridae family, such as the vesicular stomatitis Indiana virus (VSIV or VSV), the Herpes Simplex 1 virus (HHV-1 or HSV-1) and the influenza A virus (FLUAV), as well as the vaccinia virus strain Elstree (VACV) have been shown to be very sensitive to the action of O_3_-derived intermediates as reported by the drastic morphological changes of the viral capsid by electron microscopy (Murray et al., 2008). Virions without their capsid are no longer able to bind to their receptors on cells and to infect them, and are therefore unable to perform their replication cycle, resulting in a decrease in viral load.

AHT has been successfully used for the treatment of some patients with chronic hepatitis B (Cespedes-Suarez et al., 2018a). After 1 year of O_2_-O_3_ therapy, 28 patients who underwent AHT showed to be negative for the surface antigen for the surface antigens, and presented antibodies against the surface antigen itself, undetectable levels of viral load, and the restoration of normal hepatic transaminase values, which demonstrate a functional recovery of liver. Furthermore, another study from the same authors reported only a partial success in treating 32 patients with HIV-1 AIDS with O_2_-O_3_ therapy for 2 years (Cespedes-Suarez et al., 2018b). Indeed, even if the results indicated a significant decrease of viral RNA to undetectable values and an increase in the number of CD4 and CD8 lymphocytes, they found neither the restoration of immune system homeostasis nor the presence of neutralizing antibodies.

Rectal ozone therapy led to the complete remission of symptoms after 7 days in 5 symptomatic patients infected with Ebola, the hemorrhagic fever virus with high lethality (65%; Rowen et al., 2016). It has also been observed that O_2_-O_3_ therapy is able to kill SARS-CoV-1 virus in monkey cells *in vitro* and to benefit patients with bronchopneumonia and neurological complications (Li et al., 2020a; Ricevuti et al., 2020). Interestingly, SARS-CoV-2 has a sequence homology of 82% with SARS-CoV-1 (Naqvi et al., 2020) thus some authors, during the first pandemic, foresight AHT as potential treatment of COVID-19 (Ricevuti et al., 2020). Both HIV and Ebola viruses share with SARS-CoV-2 the common feature that they are coated viruses, i.e., covered by a phospholipid pericapsid, therefore they are expected to respond more effectively to the action of O_2_-O_3_ therapy, while the response of viruses with protein capsid is variable (Bocci, 1996).

Although initially uncoated viruses, such as enteroviruses, with capsid protein shell have been reported to be damaged by the action of O_3_ through peroxidation reactions (especially where there are target amino acids such as cysteine, methionine, tyrosine, histidine and lysine), other *in vivo* experiments have shown that this susceptibility is variable (Murray et al., 2008). Of note, it has been reported that type-2 adenovirus (HAdV-2), which is an uncoated virus, is more resistant to O_3_ treatment than coated viruses, therefore it seems that the inactivation degree mainly depends on the direct contact of ozone with lipids (Murray et al., 2008; Ataei-Pirkooh et al., 2021).

Lipid-coated viruses are thus highly susceptible to the action of O_3_-derived intermediates, and coronavirus belongs to this type of virus. Moreover, the S2 region of the C-terminal part of spike, which binds the human ACE2 receptor, contains a preserved area also present in all coronavirus groups (Madu et al., 2009). This area presents in SARS-coronavirus spike S2 domain, which is flanked by cysteine residues C822 and C833, is important for membrane fusion activation. This conserved area contains many cysteine residues whose thiol groups are needed to be conserved for the activation of the fusion process (Dussault et al., 1999; Rota et al., 2003; Madu et al., 2009). These thiol groups could become a second target for O_3_-derived oxidants leading to the impairment of virus entry in the host cell (Rhee et al., 2000).

Some viruses, belonging to various families including Herpesviridae, Orthomyxoviridae Rhabdoviridae, Coronaviridae, Retroviridae, Hepadnaviridae, are equipped with a pericapsid that contains, in addition to proteins and lipids, also carbohydrates. Carbohydrates are exposed outside the virion and also play a role in protecting the virus from neutralizing antibodies (Wei et al., 2003; Cenci et al., 2014). Carbohydrates are as well sensitive to the oxidizing action of O_3_-derived intermediates, and some viruses thus modified in their glycosidic pattern could be more easily recognized by the immune system (Bocci et al., 2010).

## Effect Ozone on Viruses: Action on SARS-CoV-2

SARS-CoV-2, which spread promptly around the world is a highly contagious respiratory virus with respect to SARS-CoV-1 and Middle East respiratory syndrome coronavirus (MERS) emerged in 2002 and 2012, respectively, and both showing higher mortality than SARS-CoV-2 (Hu et al., 2021). The reason is that, despite SARS-CoV-2 binds the same receptor as SARS-CoV-1, called angiotensin-converting enzyme 2 (ACE2), the mutation of 4 critical sites within the human receptor binding region (amino acids 482–485) of SARS-CoV-2 makes it even more similar and more specific in binding to ACE2 than SARS-CoV-1 (Shang et al., 2020). The SARS-CoV-2 disease has the lungs as its main target, but it is also a systemic disease because the coronavirus Spike protein binds the ACE2 receptor which is present in numerous other tissues, such as in the epithelia of the lung and small intestine as well as in arterial and venous endothelial cells and arterial smooth muscle cells of stomach, small intestine, colon, skin, lymph nodes, thymus, bone marrow, spleen, liver, kidney, and brain (Hamming et al., 2004). In severe cases, the infection produces a cytokine storm also observed in infections caused by other SARS viruses. In particular, the production of the inflammatory cytokines IL-1β, IL-6, IL-8 and TNF-α gives rise to various effects including the release of other inflammatory and chemotactic factors, which upregulate the adhesion molecules, and increases the migration of eosinophils and neutrophils, thus activating the innate response (Wang et al., 2007). Moreover, although SARS-CoV-2 gives rise to inflammatory responses phenotypically similar to other respiratory viruses, peculiar characteristics are observed especially in the dramatic increase in IL-6 and in the dysregulation of some cytokines, such as IFNs and their cytokine pathways, that could result in a poor balance between pro-inflammatory and anti-inflammatory cytokines (Wang et al., 2007; Olbei et al., 2021).

**Figure 4:**
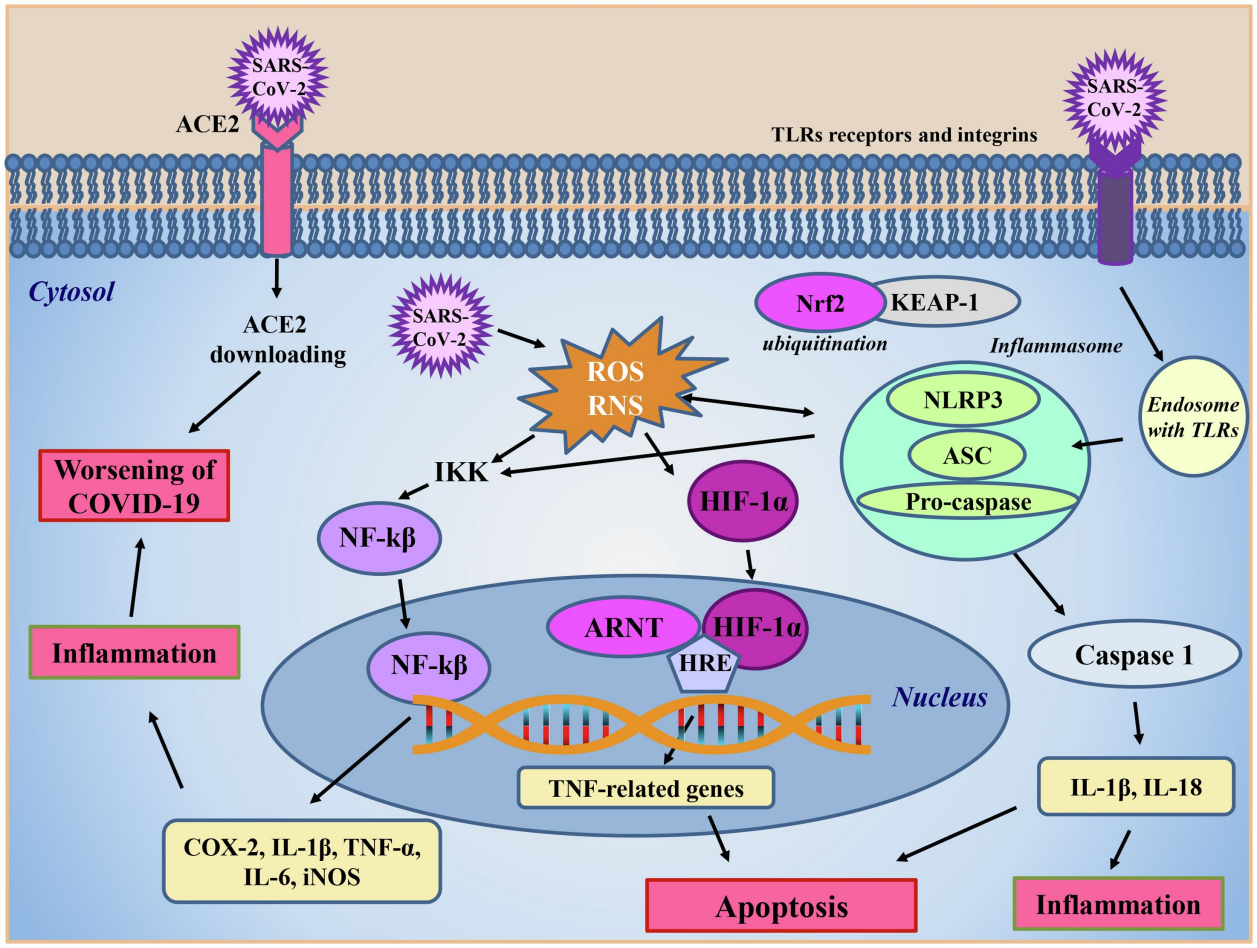
SARS-CoV-2 intracellular signaling. The main route for SARS-CoV-2 entrance in cells is through the ACE2 receptor, which subsequently undergoes to downloading. The virus enters into the cells also *via* TLR receptors or integrins. After internalization, SARS-CoV-2 promotes reactive oxygen (ROS) and nitrogen (RNS) species production and activates the inflammasome complex, formed by the nucleotide-binding domain leucine-rich repeat and pyrin domain containing receptor 3 (NLRP3), the adaptor apoptosis-associated speck-like protein (ASC) and the caspase 1 effector protein. In this condition, the nuclear factor erythroid 2-related factor 2 (Nrf2), in steady state inactivated by Keap-1 ligand, is ubiquinated favoring the inflammatory response caused by the presence of to the virus. In the presence of the inflammatory response caused to the virus, the reactive oxidizing species produced, and the activated inflammasome triggers the activation of IκB kinase (IKK) complex boosting the nuclear factor kappa-light-chain-enhancer of activated B cells (NF-kB) pathway, which in turn, translocating into the nucleus, promotes the transcription of pro-inflammatory cytokines (IL-1β, IL-6, and TNF-α) and enzymes, such as cycloxygenase (COX) and the inducible form of nitric oxide synthase (iNOS) amplifying oxidative stress and inflammation. Increased reactive oxidant species levels activate the hypoxia inducible factor 1α (HIF-1α) which, once translocated into the nucleus, interacts with the aryl hydrocarbon receptor nuclear translocator (ARNT) protein, in a promotor region hypoxia response element (HRE) on DNA, promoting TNF-related death genes transcription and ultimately leading to apoptosis. NLRP3 inflammasome activation leads to the boosting of the caspase cascade contributing to inflammation and apoptosis worsening the symptoms of COVID-19 disease.

Interestingly, peripheral blood monocytes and dendritic cells, which link the innate and adaptive response, lack the ACE2 receptor (Song et al., 2020), so they are not expected to be infected by the virus. However, IL-6 and TNF-α production by monocytes/macrophages and dendritic cells was also observed in SARS1 infection (Song et al., 2020). Therefore, it has been hypothesized that SARS-CoV-2 could enter cells through receptors others than ACE2, such as Toll-like receptors (TLR) and integrins (Figure 4). According to the data reported by Wang et al. (2007), SARS-CoV-2, through the Spike protein might interact with TLRs on the cell membrane of monocytes/macrophages and dendritic cells (Figure 4). The interaction between TLRs and Spike protein triggers the NF-kB intracellular pathways involved in inflammation, which leads to the production of TNF-α and IL-6, triggering NF-kB activity and further increasing and exacerbating the inflammatory response through an autologous/paracrine mechanism (Chirumbolo et al., 2021c; Figure 4). In addition, there is an apparent similarity between SARS-CoV-2 and metapneumoviruses host cell receptors “folding” structures, a small alpha helix loop is inserted inside two beta sheets. It has thus been hypothesized that SARS-CoV-2 could use an alternative route to enter the cell represented by integrins (Sigrist et al., 2020). Spike protein of SARS-CoV-2 has a small RGD subdomain, formed by arginine, glycine and aspartic acid, within the viral Spike receptor binding domain (amino acids 319–541 of the Spike), which is known to bind integrins. This integrin receptor is absent in all coronaviruses except metapneumoviruses, which use integrin as alternative receptor to entry into host cells (Sigrist et al., 2020). A big issue of SARS-CoV-2 with respect to SARS-1 is that the virus can enter cells through different receptors: ACE2, TLR7 and integrins (Figure 4) and other potential receptors such as glucose-regulated protein 78 (GRP78), transferrin receptor, AXL, kidney injury molecule-1, and neuropilin 1 (Dai et al., 2021). Despite the availability of different receptors, it could be speculated that ozone therapy, damaging the viral capsid, may hinder viral entry and prevent cell infection in any case.

Another big issue in COVID-19 intensive care unit (ICU) hospitalized patients is the severe hypoxia due to high level of oxidative stress and inflammation. HIF-1α is an ICU molecular oxygen sensor that in physiological condition is hydroxylated into cytoplasm and degraded *via* ubiquitin pathway. In pathological conditions, it translocates into the nucleus where it interacts with hypoxia response element (HRE) region on DNA to activate the transcription of TNF genes inducing apoptosis (Yang et al., 2017; Chirumbolo et al., 2021c). Low oxygen level increases SARS-CoV-2 infection, destabilizing the immune system in an autocatalytic vicious circle with fatal risk. In more detail, on elevated hypoxia conditions, the high level of HIF-1 α increases ACE1 and downregulates ACE2 receptors, also because of the impairment of ACE2 due to SARS-CoV-2 infection, developing the complications of disease (Li et al., 2020b; Figure 4).

In this situation, the O_2_-O_3_ AHT may have a role in immune system rebalance and prevention of endothelial damage and vascular thrombosis by the HO-1 production, *via* Nrf2 activation. Indeed, the Nrf2 induced by LOPs could boost HO-1 synthesis which, together with the production of NO, mediated by the Nrf2-activated endothelial NOS, could stabilize HIF-1α in a regulated cross-talk (Chirumbolo et al., 2021c). Moreover, the HO-1 induced by LOPs inhibits thrombosis mechanism downregulating platelet activation (Peng et al., 2004; Li et al., 2020b; Figure 4). Furthermore, the deregulation of the HO-1 seems to be associated to a more aggressive phenotype of SARS-CoV-2 (Tsiambas et al., 2020). Within the endothelium, the action of alkenals has also been related to an increase in the release of NO due to the augmented expression of the inducible isoform of nitric oxide synthase (iNOS), as observed in type II rat pneumocytes (Punjabi et al., 1994). It can be speculated that NO may have antiviral properties against SARS-CoV-2, because (i) it inhibits the palmitoylation of the Spike protein, thus preventing the attachment of the virus to the ACE2 receptor and (ii) directly or through the action of its derivatives, could cause a decrease in viral RNA production due to an effect on virus proteases (Åkerström et al., 2009; Robba et al., 2020).

Another mechanism that could lead to the hypoxia phenomena in COVID-19 could be linked to its non-structural proteins. Recently, by means of docking studies, it has been hypothesized that the non-structural proteins ORF1ab, ORF10 and ORF3a could interact with the β chain of hemoglobin by dissociating the iron atom from porphyrin (Liu and Li, 2020; Wenzhong and Hualan, 2020). In their study, the authors hypothesize that deoxyhemoglobin is more vulnerable to this attack than oxidized hemoglobin, and this attack could cause a loss of hemoglobin, producing symptoms of a respiratory crisis. This reaction could result in the decrease of hemoglobin concentration, lowering of oxygen saturation, accumulation of iron and CO_2_ in the blood of patients, increase of the levels of C-reactive protein and albumin, all events allowing to tissue toxicity. For this reason, many doctors believe that extracorporeal membrane oxygenation of patients have these peculiar clinical characteristics which may be the reason for the low success rate in saving therapy (Mustafa et al., 2020).

In addition to its antioxidant activity aimed at decreasing oxidative stress, O_2_-O_3_ therapy could improve the metabolism of erythrocytes and O_2_ supply to hypoxic tissues by improving glycolysis with the formation of 2,3-diphosphoglyceric acid (2,3-DPG) and ATP, increasing Krebs cycle and leading to the shift the HbO_2_ dissociation curve to the right (Giunta et al., 2001). These mechanisms might account for the improvement of O_2_ delivery monitored in peripheral obstructive arterial disease after O_2_-O_3_ therapy (Giunta et al., 2001). Moreover, LOPs could act as oxidative stressors in the bone marrow microenvironment by activating the release of metalloproteinases, and in particular MP-9, this could favor the activation of stem cell precursors in the marrow and peripheral blood providing neovascularization and tissue regeneration (Bocci, 2006b; Neri et al., 2017). During AHT, a dose-dependent increase in platelet activation was observed in patients, thanks to the production of their growth factors (Bocci et al., 1999).

By virtue of the above-mentioned biological and biomolecular mechanisms, numerous clinical studies have been proposed to evaluate the effects of AHT with ozone in COVID-19 disease (Table 1). Considering its wide spectrum of action and all its properties ozone therapy in the pandemic period has been used on about 50 hospitalized COVID-19 patients, all suffering from acute respiratory syndrome (ARDS), they were males over the age of 60 and subjected to other pharmacological treatments and non-invasive mechanical ventilation in intensive care (Franzini et al., 2020). In these studies, after 4 cycles of ozone therapy, with 45 μg/ml of ozone in O_2_-O_3_ mixture, in about 10 days a significant reduction of the levels of inflammatory and thromboembolic markers (CRP, IL-6, D-dimer) was observed. Notwithstanding these promising results, these studies were conducted as observational and not case–control ones (Franzini et al., 2020). In a recent study, Araimo et al. (2021) evaluated the use of O_2_-O_3_ therapy as an adjuvant support in the treatment of COVID-19. The prospective randomized study enrolled 28 patients undergoing non-invasive air-ambient ventilatory support or venti-mask (Venturi’ s mask) divided as follow: 14 in the AHT group and 14 in the control group. The procedure consisted of a double treatment with systemic O_2_-O_3_ administered standard care protocols for 7 days at a dosage of 30 μg/ml of ozone in O_2_-O_3_ mixture. Both arms were treated with standard protocols care. In this study, no statistically significant differences were found between the two groups for most of the parameters evaluated, although in the group subjected to ozone therapy, a better progression in weaning of oxygen use compared to the control group was monitored after 7 days from the treatment. The limit of this study, in addition to the low sample size, was however the reduced concentration of ozone used compared to a previous work conducted by Franzini et al. (2020). In a recent study, Fernández-Cuadros et al. (2021) evaluated the effects of rectal ozone therapy in severe COVID-19 pneumonia and compared it to the standard protocols of care (SOC) in a case control study of 28 patients subdivided in two homogeneous groups according to age, comorbidities, O_2_ saturation, and O_2_ supply. Ozone protocol consisted of eight sessions (one session/day) of intra-rectal ozone (150 ml volume, 35 μg/ml concentration corresponding to 5.25 mg O_3_ total dose). They found that the use of rectal ozone improved O_2_ saturation, reduced O_2_ supply, decreased inflammation biomarkers (fibrinogen, D-dimer, urea, LDH, CRP, and IL-6), and significantly improved radiological outcomes, when compared to the standard of care group (Fernández-Cuadros et al., 2021). Mortality and length of stay were reduced in the ozone group, but this difference was not significant. Fernández-Cuadros et al. (2020) previously found, in an observational study performed on four patients with severe pneumonia, that treatment with rectal ozone could be safe and effective hypothesizing that ozone therapy could be considered a simple alternative capable of acting on the SARS-CoV-2 virus, or an adjunctive therapeutic option to consider in the management of severe bilateral COVID-19 pneumonia. Hernández and co-workers studied, in a prospective case–control study, 18 patients with COVID-19 severe pneumonia, treated with either ozone AHT or standard treatment (Hernández et al., 2021). Patients in the treated group received ozonated blood twice daily starting on the day of admission for a median of 4 days. Each treatment involved administration of 200 ml autologous whole blood enriched with 200 ml of O_2_-O_3_ mixture with a 40 μg/ml ozone concentration. The authors concluded that O_2_-O_3_ AHT was associated with a significantly shorter time to clinical improvement in this prospective case–control study (Hernández et al., 2021). The limit of these studies is the small sample size and the study design. All results require further evaluation in larger randomized controlled trials (Fernández-Cuadros et al., 2020; Hernández et al., 2021). An Indian randomized control trial (Shah et al., 2021) involved 60 patients with mild to moderate COVID-19 disease clinically classified with a score <8 as reported by the National Early Warning Score (NEWS) which included the evaluation of clinical parameters such as the respiration rate, the blood pressure, the oxygenation rate (SpO_2_), the heart rate (Jones, 2012). The patients were divided in two parallel groups (*n* = 30/group). The interventional group (OZ) received ozonized rectal insufflation and minor AHT, as reported by Hu and co-workers (Hu et al., 2018) associated with SOC daily, while the control group (standard trial) received SOC alone. The main outcome measures included changes in clinical features, NEWS score, RT-PCR, inflammatory markers, requirement of advanced care, and metabolic profiles. The OZ group showed clinically significant improvement in the mean values of all the parameters. Ozone therapy when integrated with SOC could improve the clinical status and rapidly reduce the viral load compared to SOC alone (Shah et al., 2021). Many of the studies carried out during the first pandemic wave were observational and case reports, because this therapeutic tool was used purely for compassionate use and in advanced stages of the disease. Similarly, in randomized and controlled cases studies, such treatment was used at an advanced stage of the disease, and this represents a limit. In consideration of the biological and biochemical mechanisms described above, further studies should be better designed. Randomized control/case studies, enrolling a larger cohort of patients, are requested, using O_2_-O_3_ therapy in the early stage of disease, in particular before the evident effects of the cytokine cascade. This earlier stage can better detect the antioxidant effect, the immune regulation, and the potential effects on coagulation cascade of ozone therapy in clinical practice. It might be useful to evaluate the combined use of AHT and some polyphenols (e.g., quercetin), with similar anti-aggregating, antioxidant and immunomodulating effects. There is evidence that some nutraceuticals such as quercetin appear to act similarly against human cells and human cancer cells, reducing the levels of IL-6, IL-8 and VEGF, as described by Barbarisi et al. (2018).

**Table 1:** Summary of main clinical studies of ozone therapy in COVID-19 patients.

References	Study type	Patients enrolled	Ozone concentration and protocol	Results	Disease stage
Franzini et al., 2020	Case study	Fifty hospitalized patients. All underwent SOC+ AHT	AHT 200 ml 45 μg/ml O_2_-O_3_ 4 cycles of mixture of O_2_-O_3_ once a day for 5 consecutive days	A significant reduction of inflammatory and thromboembolic markers (CRP, IL-6, D-dimer), amelioration in the major respiratory indexes (respiratory and gas exchange markers: SpO_2_%, PaO_2_/FiO_2_ ratio).	Patients in ICU
Araimo et al., 2021	Case control study	Fourteen (O_2_-O_3_ + SOC) VS 14 (control group: only SOC)	AHT 30 μg/ml O_2_-O_3_ twice/day for 7 consecutive days	Reduction after treatment of IL-6, CRP, D dimer	All patients had ventilatory support or venturi mask
Fernández-Cuadros et al., 2021	Case control study	Fourteen (rectal ozone + SOC) VS 14 (SOC)	Rectal ozone (150 ml) concentration 35 μg/ml; for eight sessions (one session/day)	Improved O_2_ saturation and decreased O_2_ supply. Lymphocyte count improved (*p* < 0.05). Biomarkers of inflammation (fibrinogen, D-dimer, urea, ferritin, LDH, CRP, and IL-6) decreased (*p* < 0.05). Radiological findings improved (*p* < 0.0001). Mortality and LoS were inferior in the O_3_ group.	Severe COVID-19-19 pneumonia
Fernández-Cuadros et al., 2020	Case reports	Four cases	Rectal ozone (100 ml) concentration of 35 μg/ml for 5–10 days,	Reduction after treatment of IL-6, CRP, D dimer, SpO_2_% and LDH	Severe bilateral pneumonia
Hernández et al., 2021	Prospective case control study	Nine AHT+ SOC (beginning the day of admission) VS 9 only SOC (control group).	AHT 200 ml of O_2_-O_3_ mixture. Concentration: 40 μg/ml; twice per day, for 4 days	Shorter time to clinical improvement	Severe pneumonia
Shah et al., 2021	RCT	Thirty (rectal insufflation and minor AHT + SOC) VS 30 control group (only SOC)	Rectal ozone 150 ml concentration 40 μg/ml about twice daily +2–3 ml venous blood along with 5 ml ozone at 25 μg/ml [minor AHT] once daily for 10 days	Significant improvement in the mean values of all parameters tested (SpO_2_, LDH, ferritin, CRP); changes in clinical symptoms (*p* < 0.05) and inferior requirement for intensive care (*p* < 0.05).	Sixty patients with mild to moderate COVID-19 19

AHT, auto-hemotherapy; ICU, intensive care unit; SOC, standards of care; CRP, C reactive protein; LDH, lactate dehydrogenase; SpO_2_%, percentage of oxygen saturimetry; LoS, length of stay; and RCT, randomized controlled trial.

## Ozone and Microbiota

Numerous studies demonstrated that lungs of healthy people harbor a microbiota, and it is influenced by microbial signals from distal body sites, such as the intestine (Hilty et al., 2010; Bassis et al., 2015; Segal et al., 2016; Pattaroni et al., 2018).

**Figure 5:**
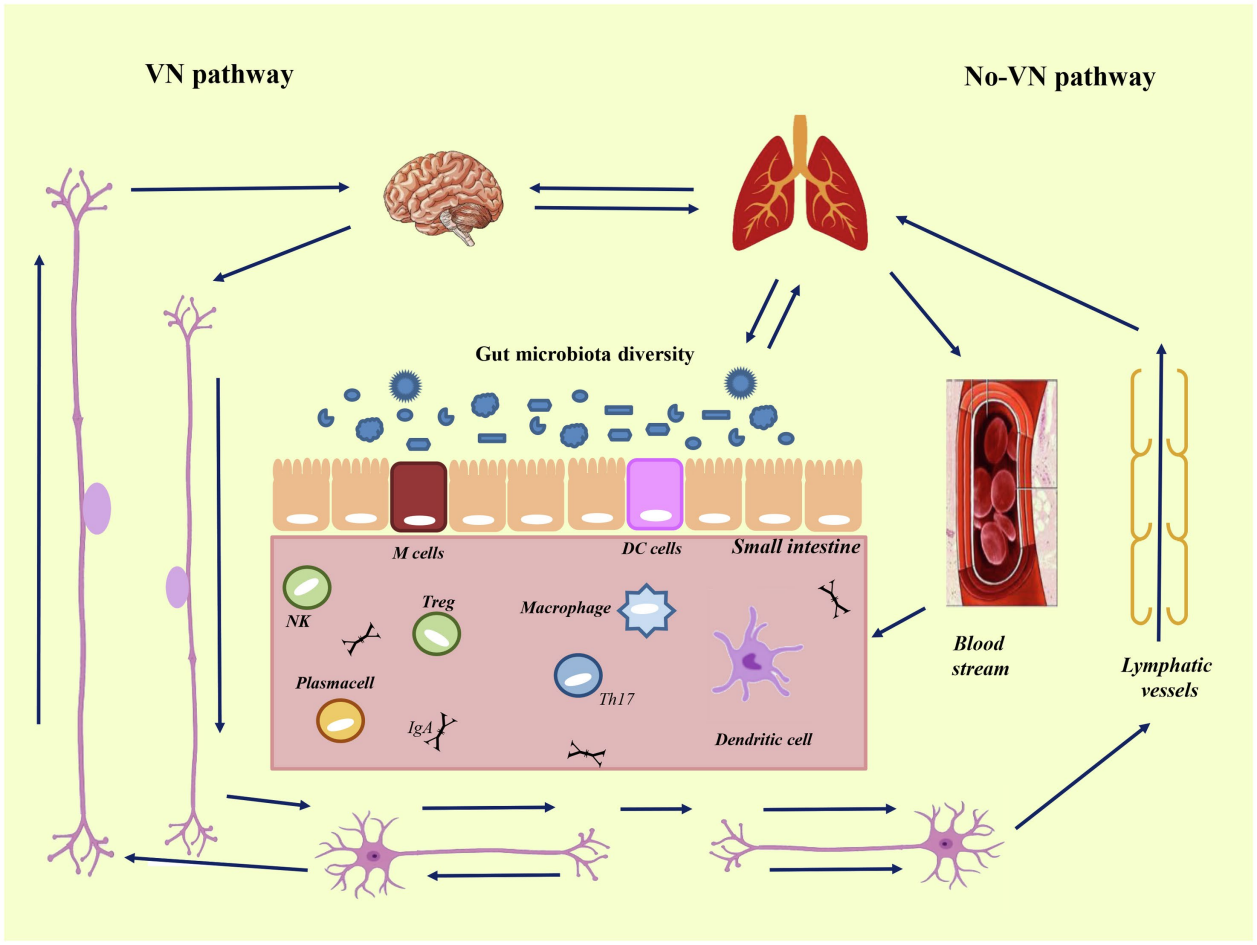
Intestine-lung-brain axis. Bidirectional axis between intestine, lung, and brain. Axis mean the pathways and cross-talks that occur between distal body sites, such as intestine, brain, and lung. The cross-talk takes place through blood stream and *via* lymphatic vessels (*via* non-VN-pathway), and *via* autonomic nervous system (*via* the vague-nervous). To the left is reported the vague-nervous pathway (VN-pathway), and to the right the non-VN-pathway, that is composed by blood stream and lymphatic vessels. The communication routes involve the autonomic nervous system (enteric nervous system and vagus nerve) and the immune system linked to the neuroendocrine system. These connected systems can affect each other in particular when the immune system, underlying the gut epithelium, became activated. Modified from Santos et al. (2019).

**Figure 6:**
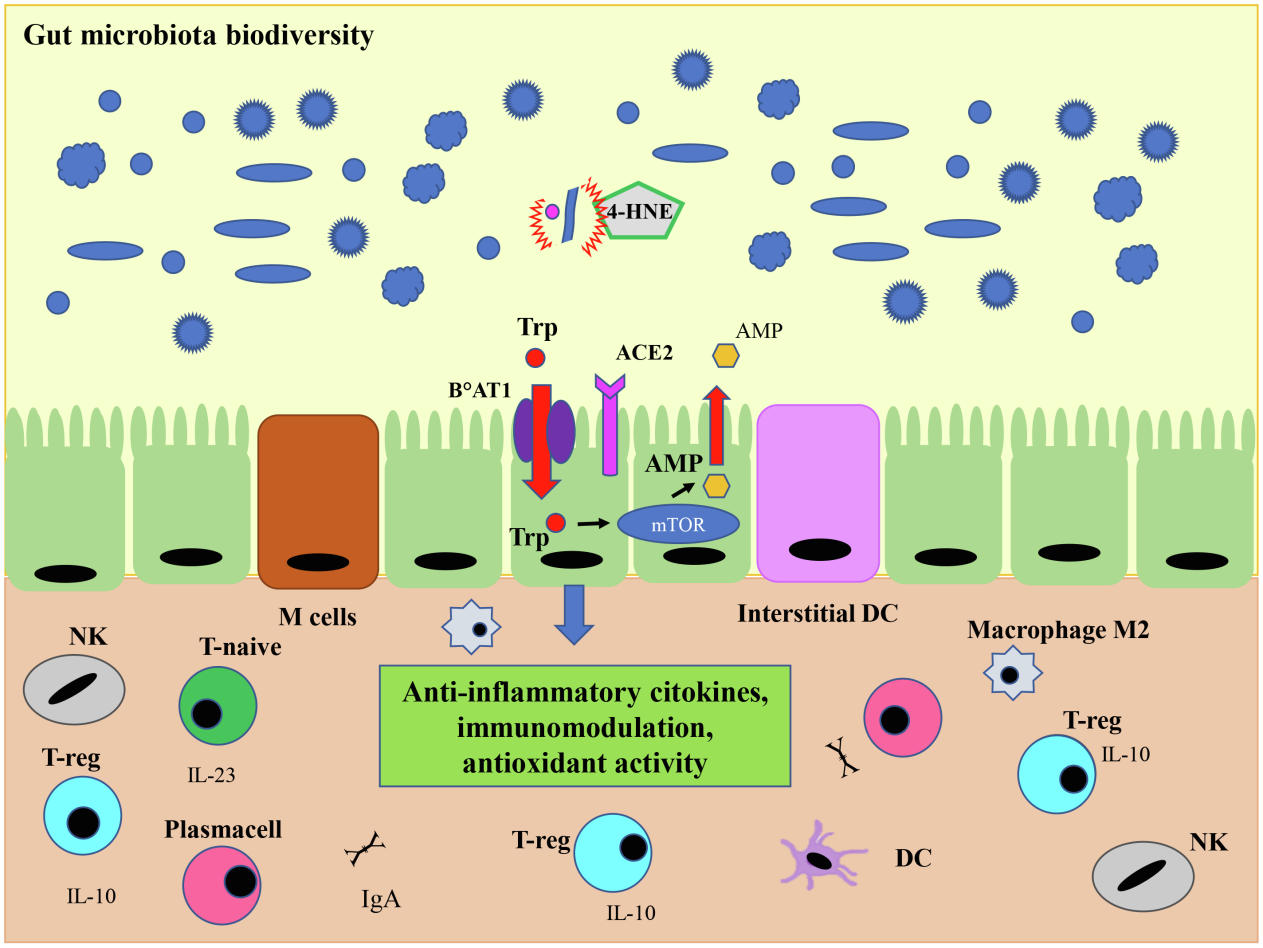
O_3_ antiviral activity and microbiota. The antiviral action mediated by ozone-derived 4-HNE leads to the viral load decrease and to a regulated expression of ACE2 on the cell membrane, important for the expression of the protein transporter of amino acid Trp (B°AT1). This transporter is metabolized by the mammalian target of rapamycin (mTor) pathway which leads to the production of the antimicrobial peptide (AMP), fundamental for immunological tolerance mediated by the regulatory T cells (T-reg). This activity results in a regulated expression of anti-inflammatory cytokines, such as IL-10 and its pathway, which in turn affects the intestinal microbiota leading to greater tolerance and therefore to a higher microbiome biodiversity. Inside the gut, both the dendritic cells and M Cells, which are some specialized epithelial cells, play a role in recognizing commensal or pathogenic bacteria. In the presence of commensal bacteria, dendritic cells secrete IL-23, stimulating the differentiation of T-naive into T-reg cells which in turn produce the anti-inflammatory cytokine IL-10. Finally, the ozone, could favor tissue repair by restoring macrophages M2 switching. Modified from Geuking et al. (2014).

These recent discoveries highlighted the main communication routes underlying the gut-lung axis (Wypych et al., 2019; Figure 5). It is well known that intestinal disbiosis is a key element in the pathogenesis of various diseases, not only affecting the intestines, but also other organs. For example, intestinal microbial disbiosis increases the risk of asthma, chronic obstructive pulmonary disease (COPD) and other respiratory infections (Budden et al., 2017; Cho et al., 2018; Voynow and Hendricks-Muñoz, 2018). In fact, there is a bowel-lung axis that concerns the potential transfer of microbes from the intestine to the lung, which is bidirectional in a way that endotoxins and microbial metabolites can affect the lung through blood and inflammation in the lung but can also affect the gut microbiota and vice versa (Figure 5). It would therefore seem that after alveolar inflammation associated with TNF-α, or in the presence of increased systemic inflammation, the pulmonary microbiome moves to acquire intestinal microbial species (Dickson et al., 2016; Voynow and Hendricks-Muñoz, 2018). In fact, it has been observed that in COVID-19 patients there is an altered and weakened intestinal microbiome, characterized by an important decrease in biodiversity, determined by a reduction in symbiotic beneficial bacteria populations and an enrichment of opportunistic pathogens (Streptococcus, Rothia, Actinomyces), which may be related to gastrointestinal disorders (e.g., abdominal pain, nausea, vomiting, and diarrhea) of the acute phase of infection (Ferreira et al., 2020; Gu et al., 2020; Zuo et al., 2020). These symptoms are often reported at the onset of SARS-CoV-2 infection, and might precede respiratory disorders (Villapol, 2020). There is therefore a correlation between the severity of the symptoms associated with COVID-19 and the composition of the fecal microbiota. It is known that SARS-CoV-2 infects the intestinal epithelium cells that are extremely rich in ACE2 receptors. When the microbiota in a healthy patient is in balance, there is an immune tolerance towards the antigens derived from its inner organisms, which promote the differentiation of T-reg cells by dendritic cells and stimulate the secretion of anti-inflammatory cytokines, such as IL-10 (Kamada et al., 2013; Figure 6). Otherwise, the presence of pathogenic agents, can trigger pro-inflammatory immune responses due to differentiation of naïve T lymphocytes into Th1 and Th17 cells.

**Figure 7:**
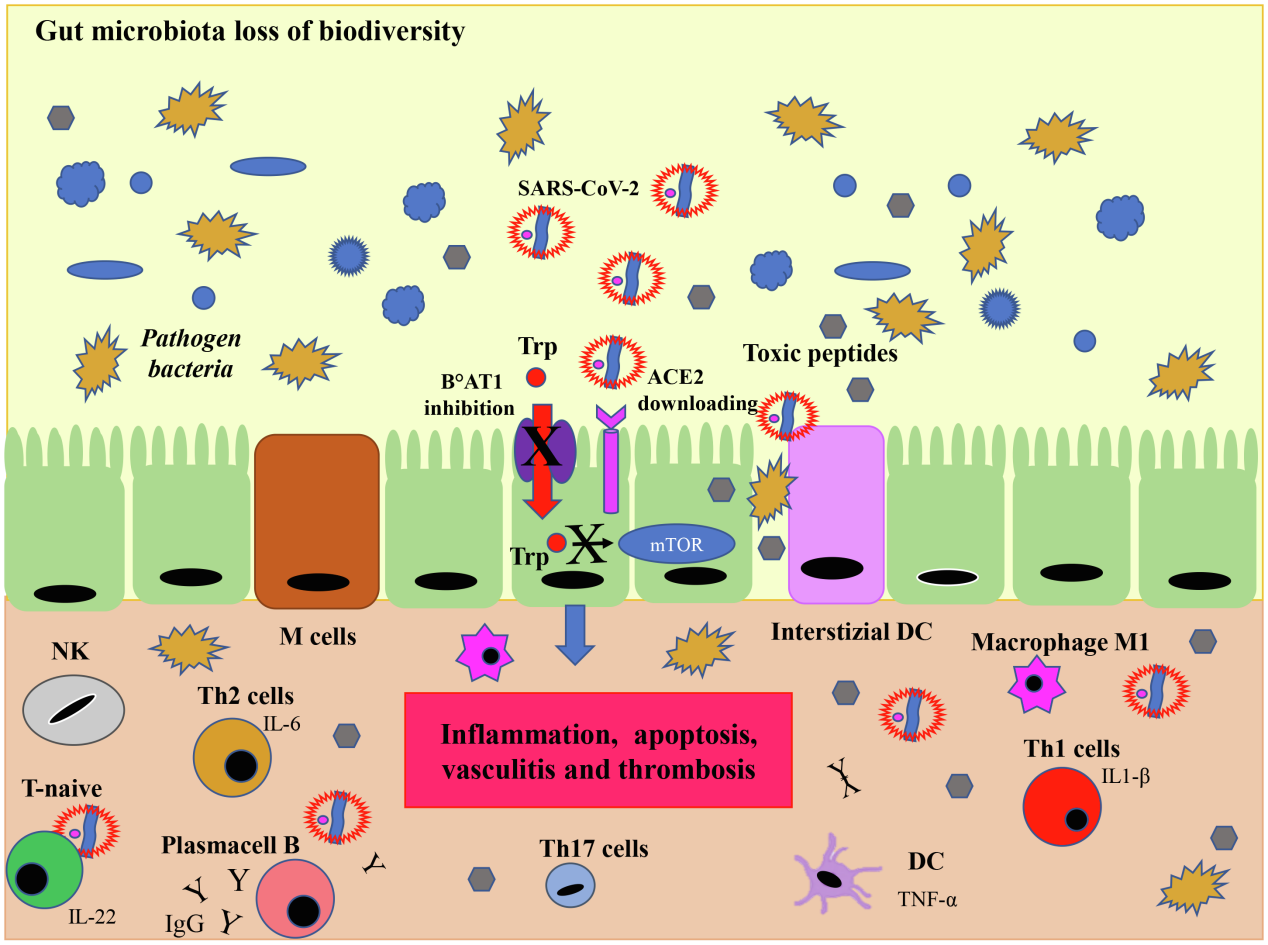
SARS-CoV-2 infection and microbiota. Although the viral mechanism of action in microbiota is not fully understood, the downregulation of ACE2 induced by SARS-CoV-2 infection limits the activity of the mammalian target of rapamycin (mTOR) pathway by reducing the absorption of tryptophan. This process results in a reduced secretion of the AMP which in turn, due to the loss of immunological tolerance, leads to dysbiosis, i.e., a reduced biodiversity of eubacteria and an increase in pathogenic bacteria which release toxic peptides. In this dysbiotic environment the production of IL-17 and IL-22, induce the differentiation of Th1 and Th17 cells which release pro-inflammatory cytokines. In this case, interstitial dendritic cells (DC) and gut specialized M cells produce inflammatory cytokines, such as TNF-α which transform T naïve cells into IL-1β secreting T cells (Th1) and in IL-17 producing T cells (Th17) mediating the inflammatory response with activation of inflammatory macrophages M1. T helper 2 stimulate B cells to produce IgG and IgA. The downregulation of ACE2 leads to a series of negative cascade effects including an increased inflammation resulting in damage to the tight junctions of the intestinal barrier, apoptosis, vasculitis and thrombosis. Modified from Geuking et al. (2014).

Although the mechanisms by which SARS-CoV-2 can create disbiosis are not fully understood, it is known that the downregulation of ACE2 due to viral infection reduces intestinal absorption of tryptophan, which in turn reduces the activity of the mTOR pathway in the small intestine. The consequent decrease in the secretion of antimicrobial peptides (AMPs) results in pathogens increased survival and intestinal disbiosis (Liévin-Le Moal and Servin, 2006; Kamada et al., 2013; He et al., 2020; Ranaldi et al., 2020), thus altering the composition of the gut microbiota (Figure 7). Summing up, ACE2 would also have a role in modulating innate immunity and influencing the composition of the gut microbiota. The intestine is a particularly vascularized body organ rich in lymphocytes called gut-associated lymphoid tissues (GALTs), for their normal maturation microbiome is required (Kamada et al., 2013). Inside the gut, both the dendritic cells and M cells, which are specialized epithelial cells, play a role in recognizing commensal or pathogenic bacteria. In the presence of commensal bacteria, dendritic cells secrete IL-23 to stimulate the differentiation of T-naive into T-reg cells which in turn produce the anti-inflammatory IL-10 cytokine, whereas in a dysbiotic environment the production of IL-17 and IL-22, stimulate the differentiation of Th1 and Th17 cells which release pro-inflammatory cytokines (Barko et al., 2018; Mönkemüller et al., 2020). Alterations in the gut microbiome together with lymphocytes production of inflammatory cytokines, also sustained by viral infection, can lead to the vasculitis and thrombosis observed in patients with COVID-19 (Mönkemüller et al., 2020; Ranaldi et al., 2020; Figure 7). Although there are currently few studies on the effects of O_3_ on the microbiome, the preliminary results deserve interest and further study. In a paper on atypical dermatitis caused by *Staphylococcus aureus*, which is present at 90% of the microbiome in these lesions, treatment with topical ozonized oil not only reduced the amount of *S. aureus* due to its bactericidal effect, but significantly restored microbiological diversity (*p* < 0.05; Zeng et al., 2020). An explanation for these data could be that the significant increase in the conditional pathogen Acinetobacter Ropheus could stimulate the production of interleukin 10 by monocytes and keratinocytes modulating the balance of Th1/Th2 cells differentiation and exerting an anti-inflammatory effect (Fyhrquist et al., 2014). IL-10 is also one of those anti-inflammatory cytokines that has been associated with the immunomodulatory action of ozone therapy. Another cytokine produced by both ozone and microbiota protecting from respiratory infections is GM-CSF through interleukin-17A. Interleukin 17A displays an action on both innate and adaptive immune response, and it is involved in the host’s antimicrobial defense and tissue integrity maintenance (Boisson et al., 2013).

Another important immunomodulatory effect of ozone in the blood is the induction of macrophages phenotypic switch from the inflammatory M1 to anti-inflammatory M2 phenotype (Figure 6). Indeed, macrophages through the expression of receptors such as sterol regulatory element binding protein (SREBP) and liver X receptor alpha (LXRα) could activate the inflammatory or anti-inflammatory pathways, respectively, in response to cellular signaling and the cytokines released in the microenvironment (Chirumbolo et al., 2021c). In this way ozone, by restoring macrophages M2 switching, could favor tissue repair. Ozone, therefore, thanks to its immunomodulating capabilities is an adjuvant for the restoration of homeostasis and could have positive effects on the microbiota.

## Environmental Ozone and SARS-CoV-2

It is worth to mention that as already pointed out in the introduction, O_3_ has effects other than the medical use described so far, such as its use as a gas for environmental disinfection. Recent studies reported a role for gaseous ozone in the disinfection of surfaces from SARS-CoV-2 (Yano et al., 2020; Albert et al., 2021; Criscuolo et al., 2021; Percivalle et al., 2021). This anti-viral action of O_3_ occurs outside the human organism and deals with O_3_ used as disinfectant in environmental spaces and indoor areas. In addition, it is well known that the presence of ozone in troposphere can be harmful. Recently, it has been reported that air pollution strongly correlates to increased SARS-CoV-2 morbidity and mortality (Liang et al., 2020; Wu et al., 2020). In particular Vo’s group (Vo et al., 2022) demonstrated for the first time the relations between ground-level ozone and the susceptibility of individuals to SARS-CoV-2. These authors investigated the impact of ozone inhalation on the expression levels of genes associated with host susceptibility to SARS-CoV-2 in lung tissues collected from mice sub-chronically exposed to 0.8 ppm ozone or filtered air, as a vehicle, for 3 weeks. The reported results showed that as ozone exposure increased the two critical SARS-CoV-2 host susceptibility genes [i.e., those related to ACE2 and to the transmembrane protease serine 2 (TMPRSS2)] expression rose in parallel. TMPRSS is a protease generated by host cells capable of priming Spike protein to activate the binding of the SARS-CoV-2 to ACE2. Taken together, these results show that ozone environment pollution might increase the risk of severe respiratory illness not only with regard to SARS-CoV-2, but also to other respiratory viruses, such as SARS-CoV-1, MERS, and influenza A, in which TMPRSS2 is essential for the proteolytic processing (Vo et al., 2022).

## Conclusion

In conclusion, it is clear that the effects of ozone occur by different mechanisms. First of all, there is a O_3_-derived intermediates action on viruses through the oxidation of the capsid, with more efficiency in the lipid coated viruses than in those with the protein capsid, which drastically reduces the viral load. The second mechanism relates exclusively to the therapeutic concentrations of the mild oxidative action with reactive oxidizing species production and prompt expression of inflammatory cytokines, by means of NF-Kβ. The third effect is the formation of alkenals, due to the oxidation of fatty acids in the blood, in particular Omega 6 and Omega 3 transported by albumin. They act as signal transducers by activating the cytoplasmic protein Nrf2, and therefore the transcription of several antioxidant genes under the ARE promoter. These gene products inhibit the cascade of inflammatory cytokines, and the NRLP3 inflammasome, and lead to the synthesis of anti-inflammatory cytokines such as IL-10. The ozone changes in cytokine activation pathways from pro-inflammatory initially to anti-inflammatory immediately afterwards have a direct effect on the activation of regulatory T lymphocytes, with immunoregulatory action both on the immune system and on the microbiota. The latter is increasingly considered as a barrier and balance element, since the intestinal microbiota can influence the immune response thus regulating the progression of the disease, acting both on the innate and adaptive immune arms. Especially in this historical period where there is an increasing risk of emerging viral infections, it could be useful to design clinical trial on larger cohorts of patients with the aim to find effective ozone concentrations in the therapeutical setting, but also to understand the role of O_2_-O_3_ therapy in the earlier stage of disease and to deeply explore all the possible biological effects of this treatment.

## Author Contributions

AC, VD, DP, IM, and VS reviewed the literature, contributed to writing, and developed and edited the manuscript, the table and the figures. CS contributed to reviewing the manuscript and editing the manuscript. All authors contributed to the article and approved the submitted version.

## Conflict of Interest

CS is employed by APS S.p.a.

The remaining authors declare that the research was conducted in the absence of any commercial or financial relationships that could be construed as a potential conflict of interest.

## Publisher’s Note

All claims expressed in this article are solely those of the authors and do not necessarily represent those of their affiliated organizations, or those of the publisher, the editors and the reviewers. Any product that may be evaluated in this article, or claim that may be made by its manufacturer, is not guaranteed or endorsed by the publisher.
